# Clinical and radiographic response following targeting of BCAN-NTRK1 fusion in glioneuronal tumor

**DOI:** 10.1038/s41698-017-0009-y

**Published:** 2017-03-20

**Authors:** Christopher Alvarez-Breckenridge, Julie J. Miller, Naema Nayyar, Corey M. Gill, Andrew Kaneb, Megan D’Andrea, Long P. Le, Jesse Lee, Ju Cheng, Zongli Zheng, William E. Butler, Pratik Multani, Edna Chow Maneval, Sun Ha Paek, Brian D. Toyota, Dora Dias-Santagata, Sandro Santagata, Javier Romero, Alice T. Shaw, Anna F. Farago, Stephen Yip, Daniel P. Cahill, Tracy T. Batchelor, A. John Iafrate, Priscilla K. Brastianos

**Affiliations:** 1 Department of Neurosurgery, Massachusetts General Hospital, Harvard Medical School, Boston, MA USA; 2 Department of Neurology, Massachusetts General Hospital, Harvard Medical School, Boston, MA USA; 3 Department of Medicine, Massachusetts General Hospital, Harvard Medical School, Boston, MA USA; 4 Department of Pathology, Massachusetts General Hospital, Harvard Medical School, Boston, MA USA; 5 Ignyta, Inc., San Diego, CA USA; 6 Department of Neurosurgery, Seoul National University Hospital, Seoul, Korea; 7 Division of Neurosurgery, Department of Surgery, University of British Columbia, Vancouver, BC Canada; 8 Department of Pathology, Division of Neuropathology, Brigham & Women’s Hospital, Harvard Medical School, Boston, MA USA; 9 Department of Radiology, Massachusetts General Hospital, Harvard Medical School, Boston, MA USA; 10 Department of Pathology & Laboratory Medicine, University of British Columbia, Vancouver, BC Canada; 11 Division of Hematology and Oncology, Massachusetts General Hospital, Harvard Medical School, Boston, MA USA; 12 Department of Radiation Oncology, Massachusetts General Hospital, Harvard Medical School, Boston, MA USA

## Abstract

Glioneuronal tumors constitute a histologically diverse group of primary central nervous system neoplasms that are typically slow-growing and managed conservatively. Genetic alterations associated with glioneuronal tumors include *BRAF* mutations and oncogenic fusions. To further characterize this group of tumors, we collected a cohort of 26 glioneuronal tumors and performed in-depth genomic analysis. We identified mutations in *BRAF* (34%) and oncogenic fusions (30%), consistent with previously published reports. In addition, we discovered novel oncogenic fusions involving members of the *NTRK* gene family in a subset of our cohort. One-patient with *BCAN* exon 13 fused to *NTRK1* exon 11 initially underwent a subtotal resection for a 4th ventricular glioneuronal tumor but ultimately required additional therapy due to progressive, symptomatic disease. Given the patient’s targetable fusion, the patient was enrolled on a clinical trial with entrectinib, a pan-Trk, ROS1, and *ALK* (anaplastic lymphoma kinase) inhibitor. The patient was treated for 11 months and during this time volumetric analysis of the lesion demonstrated a maximum reduction of 60% in the contrast-enhancing tumor compared to his pre-treatment magnetic resonance imaging study. The radiologic response was associated with resolution of his clinical symptoms and was maintained for 11 months on treatment. This report of a *BCAN-NTRK1* fusion in glioneuronal tumors highlights its clinical importance as a novel, targetable alteration.

## Introduction

Glioneuronal tumors are a collection of uncommon, diverse primary central nervous system (CNS) neoplasms that exhibit variable degrees of glial and neuronal differentiation. The prognosis for patients with these tumors is generally favorable due to their non-infiltrative, well-circumscribed, and surgically accessible features. However, a subset of these patients have non-resectable disease or tumors that take on an unusually aggressive course, necessitating treatment with either radiation or chemotherapy, which both have limited efficacy. Glioneuronal tumors are histologically, genetically, and clinically diverse. Advances in the molecular characterization of CNS tumors, particularly in primitive neuroectodermal tumors of the central nervous system^1^ and low grade gliomas,^2^ have provided a blueprint for creating a similar molecular framework for classification of histologically heterogeneous glioneuronal tumors. In addition to refining the classification and diagnosis of a previously diverse collections of tumors, recent advances in molecular profiling has aided in the identification of novel oncogenic drivers and targetable alterations leading to expansion of targeted therapies across various cancer. These include targeting *EGFR* mutations and oncogenic *ALK* fusions in NSCLC with tyrosine kinase inhibitors,^3, 4^ and oncogenic *BRAF* mutations in melanoma and NSCLC with dabrafenib and trametinib.^5^ Thus, in the modern era, genomic characterization and drug development often progress in parallel to facilitate the rapid evaluation of novel pharmaceutical agents against newly identified, putative oncogenic drivers.^6^

## Results

To explore the diversity of genetic alterations in glioneuronal tumors, a cohort of 26 tumors with pathologic diagnoses that included glioneuronal tumor or ganglioglioma was collected (15 from Massachusetts General Hospital, Boston, 11 from Vancouver General Hospital, Vancouver), and examined for BRAF V600 and IDH1 mutations and oncogenic fusions using targeted next generation sequencing (NGS).^7^ BRAF V600E and IDH1 R132H mutant protein expression was confirmed by immunohistochemistry. As expected, we identified several recurrent *BRAF V600E* mutations (9 of 26), consistent with previous reports.^8^ In addition, we identified known and novel fusions of *FGFR1-TACC1*, *KIAA1549-BRAF*, *PATZ1-EWSR1*, *PRKAR2B-BRAF*, *STRN3-NTRK2*, *WNK2-NTRK2*, and *BCAN-NTRK1* (8 of 26) (Table 1). Of note, three tumors in our cohort contained a fusion involving the neurotrophic tropomyosin receptor kinase gene family (*NTRK)*, which encode the Trk transmembrane receptors. Fusions involving the *NTRK* family have been reported in a number of different cancers and lead to constitutive activation of Trk protein kinase activity.^9, 10^

**Table 1:** Molecular alterations found in glioneuronal tumors

Histologic diagnosis	Who grade	Age at diagnosis	Fusions	Braf V600E mutation
Glioneuronal tumor	I	33	None	No
Glioneuronal tumor	I	31	KIAA1549 ex16-BRAF ex9	No
Glioneuronal tumor		25	None	No
Glioneuronal tumor		32	None	No
Glioneuronal tumor		26	EWSR1 ex9-PATZ1 ex1	ND
Low-grade glioneuronal tumor		18	None	ND
Low-grade glioneuronal tumor		34	BCAN ex13-NTRK1 ex11	No
Low-grade glioneuronal tumor		15	FGFR1 ex18-TACC1 ex7	No
Low-grade glioneuronal tumor		30	None	Yes
Low-grade glioneuronal tumor		29	None	Yes
Low-grade glioneuronal tumor		19	None	No
Low-grade glioneuronal tumor		74	None	No
Diffuse and complex glioneuronal lesion		42	None	Yes
Glioneuronal tumor with focally elevated proliferation index		33	None	ND
Malignant glioneuronal tumor		33	None	Yes
Complex glioneuronal tumor		37	WNK2 ex24-NTRK2 ex16	No
Ganglioglioma	I	20	None	Yes
Ganglioglioma	I	33	STRN3 ex7-NTRK2 ex16	No
Ganglioglioma	I	39	PRKAR2B ex1-BRAF ex10	No
Ganglioglioma	I	70	None	No
Composite ganglioglioma/Pilocytic astrocytoma	I	24	None	Yes
Anaplastic ganglioglioma		24	None	Yes
Atypical ganglioglioma	II	33	None	No
Composite DNT and ganglioglioma		29	None	Yes
Composite ganglioglioma and DNT	I	23	None	Yes

*WHO* World Health Organization, *DNT* dysembryplastic neuroepithelial tumor, *ND* not done

Consistent with recent reports documenting targetable *NTRK* fusions in other cancers, including a small percentage of patients with non-small cell lung cancer,^11^ our finding of a *BCAN-NTRK1* fusion in a glioneuronal patient raised the possibility of therapeutic intervention. A 54-year-old man with a tumor containing this fusion initially underwent a resection of the symptomatic, enlarging 4th ventricular mass. Due to the low-grade features of the tumor and its proximity to the medulla, a subtotal resection, freeing entry of the cerebral aqueduct was performed.

**Fig. 1:**
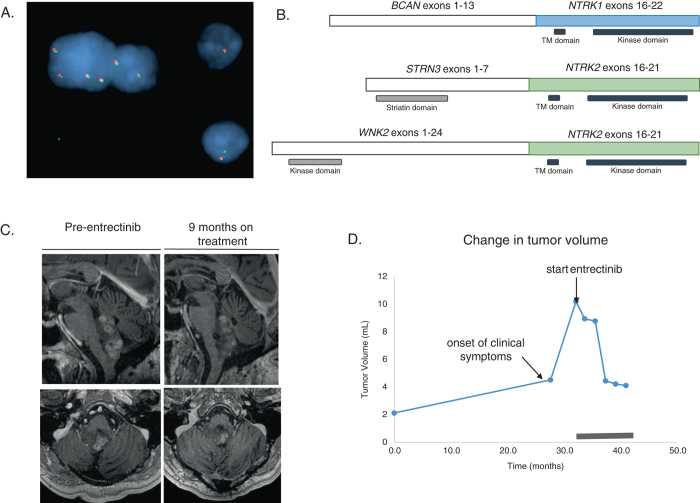
*NTRK* fusion in glioneuronal tumors can be treated with Trk-inhibitors. **a** Fluorescence in situ hybridization (FISH) using split apart probes, with separation of the 5′ (*green*) and 3′ (*red*) *NTRK1* signals, reveal abnormal rearrangement, with some *red*–*green* pairs showing a small green probe signal (*arrows*). **b** Schematic of three different *NTRK-*containing gene fusions discovered in the glioneuronal cohorts from MGH and Vancouver, involving either *NTRK1* or *NTRK2.* Predicted active domains in the expression product are depicted below. *TM* transmembrane. **c** Sagittal (*top*) and axial (*bottom*) post-contrast T1-weighted MRI images of patient with *BCAN-NTRK1* fusion just prior to treatment with entrectinib (*left*) and following 9 months on treatment (*right*). The T1-avid tumor visualized in dorsal pons and medulla has decreased in size during this time period. **d** Plot demonstrating tumor volume over time while patient was on treatment with entrectinib using MRI-derived volumetrics (see Methods). Baseline tumor volume was measured approximately 2 years prior to treatment. Tumor volume initially slowly increased, then expanded more rapidly coincident with onset of clinical symptoms. Treatment with entrectinib led to rapid and substantial decrease in tumor volume. Period on treatment denoted with *thick black line*

Findings from the permanent pathologic specimen were notable for a low cellularity tumor in a densely fibrillary background, numerous Rosenthal fibers and eosinophilic granular bodies, and a low Ki-67 labeling index (~3%). The tumor included a heterogeneous population of GFAP and synaptophysin immunopositive cells and did not stain for either NeuN or IDH1 R132H. These collective findings were felt to be consistent with a mixed low-grade glioneuronal tumor with pilocytic features. Following a period of clinical and radiologic stability, an magnetic resonance imaging (MRI) performed 3 years after the surgery revealed interval growth in the lesion with associated mass effect on the pons. Given the patient’s indolent symptom of diplopia, mild progression on imaging and reluctance for radiotherapy, targeted pharmacological therapy options were considered. Targeted NGS was performed on RNA extracted from the tumor and uncovered a fusion (confirmed by FISH, Fig. 1a), involving *BCAN* exon 13 fused to *NTRK* exon 11, including an intact and in-frame tyrosine kinase domain of TrkA (Fig. 1b). He enrolled on a phase 1 dose-escalation clinical trial of entrectinib (RXDX-101), a pan-Trk, ROS1, and ALK oral tyrosine kinase inhibitor (ClinicalTrials.gov Identifier: NCT02097810). Entrectinib was recommended given previous reports of clinical activity in tumors harboring *NTRK* gene fusions in colorectal carcinoma and non-small cell lung cancer^11, 12^ as well as for the demonstrated activity of entrectinib to penetrate the central nervous system.^11^ The patient received an entrectinib dose of 600 mg orally each day and experienced lower extremity edema as the only documented side effect of the medication.

After 9 months of therapy, volumetric analysis of the lesion demonstrated a 60% reduction in contrast-enhancing tumor compared to his pre-treatment MRI (Fig. 1c, d). This radiographic improvement was accompanied by improvement of diplopia, which incidentally worsened with temporary cessation of entrectinib for 5 days during his treatment course. Two months later, however, the patient developed worsening diplopia and imaging of the lesion demonstrated a gradual interval increase in size. Therefore, in light of radiographic progression and worsening symptoms, the decision was made to discontinue entrectinib after 11 cycles and refer the patient for radiotherapy.

## Discussion

In our cohort of 26 glioneuronal tumors, the prevalence of BRAF pathway activation (34% patients with mutations, 7% with fusion) and gene fusions involving other oncogenes (30% of patients) is consistent with recently published results across a series of low-grade neuroepithelial tumors in children, which found BRAF alterations in 9 of 17 gangliogliomas (including *BRAF V600E* mutation, *MACF1-BRAF*, *AGK-BRAF*, and *GNAI1-BRAF* fusions).^13^ In common with our cohort, Qaddoumi *et al*. also observed fusions in *EWSR1-PATZ1* and *SLMAP-NTRK2* in 2 out of 17 gangliogliomas.^13^ Moreover, fusions incorporating *FGFR1-TACC1* and *BRAF-RNF130* have been reported in dysembryoplastic neuroepithelial tumor and diffuse oligodendroglial tumors, and *KIAA1549-BRAF* has been established as a driver in infratentorial pilocytic astrocytomas.^13^ These results suggest that BRAF alterations and oncogenic fusions are key drivers in glioneuronal pathogenesis and represent a potential target for molecularly guided therapy. In the setting of glioneuronal tumors, a variety of genetic alterations, each occurring at relatively low frequency, appear to contribute to their development. Our finding of *BCAN-NTRK1, STRN3-NTRK2 and WNK2-NTRK2* fusions highlight *NTRK*-related fusions as a recurrent alteration in glioneuronal tumors.

Fusions involving *NTRK1, NTRK2*, and *NTRK3* also have been reported to occur at a low frequency across multiple tumor types.^14^ Analysis of The Cancer Genome Atlas shows *NTRK1*, *NTRK2*, and *NTRK3* fusions with concomitant oncogenic activation in multiple signaling pathways, such as MAPK and AKT, across a variety of tumors.^15^ Further, Jones *et al*. report recurrent *NTRK2* fusions in pediatric pilocytic astrocytomas^16^ and Kim *et al*. report a BCAN-NTRK1 in glioblastoma.^17^ Based on the results from our study, we propose that *NTRK* fusions are novel oncogenic events that similarly serve as actionable targets.

The utility of targeting *NTRK1* was first described by Vaishnavi *et al*. in lung cancer where the authors identified oncogenic fusions involving *MPRIP-NTRK1* and *CD74-NTRK1*, leading to constitutive activation of the kinase domain of the *NTRK1* expression product, TrkA.^18^ The relevance of this finding was demonstrated in 3 of 91 lung cancer patients with newly diagnosed *NTRK1* fusions. Targeting these fusions with ARRY-470, CEP-701, and crizotinib, which inhibit autophosphorylation of *MPRIP-NTRK1* and *CD74-NTRK1*, led to inhibited proliferation and colony formation, and induced cell cycle arrest,^18^ validating the oncogenicity of these gene fusions.

The utility of this approach was further highlighted in the context of *LMNA-NTRK1* fusions in soft tissue sarcoma, congenital infantile fibrosarcoma (CIFS), and colorectal cancer.^12, 19–21^ In the setting of metastatic soft tissue sarcoma of the thigh, targeting the lamin A/C (*LMNA*) and *NTRK1* fusion with the TrkA inhibitor, LOXO-101, led to a rapid clinical, radiographic, and serologic response.^19^ Similarly, an infant with metastatic CIFS was found to have an *LMNA-NTRK1* fusion in addition to biallelic losses of *CDKN2A* and *CDKN2B*. The child was started on crizotinib and after 6 weeks of treatment demonstrated regression of metastatic disease.^21^

The *LMNA-NTRK1* fusion has similarly been demonstrated in colorectal cancer.^12, 20^ In a study by Sartore-Bianchi *et al*. a patient with primary colon cancer, peritoneal carcinomatosis, and liver metastases was similarly found to have a *LMNA-NTRK1* fusion. Treatment with entrectinib was initiated, resulting in a partial response with decrease in the size of multiple metastatic lesions.^20^ However, the patient, who was being treated on an intermittent dosing schedule during the early stages of entrectinib dose finding, ultimately developed disease progression in the setting of treatment resistance. Interestingly, Russo and colleagues noted that, at the time of tumor progression, circulating tumor DNA was found to have two novel *NTRK1* mutations (*NTRK1*, p.G595R and p.G667C) that were not detectable in the plasma at the initiation of therapy, demonstrating evidence of acquired resistance to entrectinib.^20^

In our study, we expand upon the recent success of targeting *NTRK* fusions across various cancer types by reporting the first treatment of a glioneuronal tumor with a pan-Trk inhibitor. This treatment was associated with a radiographic and clinical response for a sustained period of time. Thus, our results underscore the importance of examining newly diagnosed glioneuronal tumors for fusions, while also emphasizing the need for ongoing drug development to target these novel oncogenic fusions. These results emphasize the value of identifying unique molecular subpopulations of patients with low-frequency genomic alterations.^6^ More generally, our findings highlight the need for a tailored approach to oncologic care in which patient samples are examined for unique molecular drivers that can ultimately be treated with emerging targeted therapies. This work also highlights the need for CNS penetrant compounds in order to effectively treat primary CNS neoplasms as well as other solid tumors with a propensity to metastasize to the brain.

## Methods

### Gene fusion assay

The Anchored Multiplex PCR for targeted fusion transcript detection using NGS was used, as previously described.^7^ Briefly, total nucleic acid was isolated from a formalin-fixed paraffin embedded tumor specimen after histological review for tumor enrichment. The total nucleic acid was reverse transcribed with random hexamers, followed by second strand synthesis to create double-stranded complementary DNA (cDNA). The double-stranded cDNA was end-repaired, adenylated, and ligated with a half-functional adapter. Two hemi-nested PCR reactions were applied to create a fully functional sequencing library that targets specific genes (exons) listed below. Illumina MiSeq 2 × 147 base pair paired-end sequencing results were aligned to the hg19 human genome reference using bwa-mem.^22^ A laboratory-developed algorithm was used for fusion transcript detection and annotation. The integrity of the input nucleic acid and the technical performance of the assay were assessed with a qualitative reverse transcription qPCR assay and assessing the DNA/RNA content in the sequencing results. The assay is validated for samples showing 20% or higher tumor cellularity. FISH was performed on a 5-µ formalin-fixed paraffin embedded tumor section, pretreated with xylene and standard protease and detergent treatment. BAC probe RP11 -1047J23 (5′ NTRK1) was labeled green and RP11 -1038N13 (3′ NRTK1) red. Images were captured with an Olympus BX61 fluorescence microscope and equipped with a Leica Cytovision workstation.

### Volumetric analysis

Volumetric analysis has been shown to be a sensitive indicator of tumor growth, particularly in tumors with complex shape or slow growth, and is widely used for monitoring changes in nervous system tumors.^23, 24^ Routine MRI containing standard imaging sequences, including T2-, FLAIR-, and T1-weighted sequences were obtained before and after administration of gadolinium. Volumetric measurements were performed with semi-automated outline on 3 mm T1-weighted post-contrast images. Vitrea, Vital Images, Minnetonka, Minnesota USA.^23–26^
